# Diagnostic Utility of Serum Neutrophil Gelatinase-Associated Lipocalin in Polytraumatized Patients Suffering Acute Kidney Injury: A Prospective Study

**DOI:** 10.1155/2018/2687584

**Published:** 2018-11-06

**Authors:** Lukas Leopold Negrin, Reinhard Hahn, Thomas Heinz, Stefan Hajdu

**Affiliations:** ^1^Department of Orthopedics and Trauma Surgery, Medical University of Vienna, Austria; ^2^Department of Anesthesiology, General Intensive Care and Pain Management, Medical University of Vienna, Austria

## Abstract

**Introduction:**

The incidence of acute kidney injury (AKI) considerably increases the mortality rate in polytrauma victims. Undoubtedly, early identification of patients at risk is crucial for timely implementation of preventive strategies in order to improve their prognosis. Therefore, we aimed to investigate if serum neutrophil gelatinase-associated lipocalin (sNGAL) may serve as a diagnostic biomarker of early AKI in polytrauma victims, especially considering patients needing renal replacement theory (RRT).

**Material and Methods:**

Forty consecutive polytrauma victims (ISS ≥ 16, AIS_Thorax_ ≥ 1, age ≥ 18 years, survival time ≥ 48 hours), directly admitted to our level I trauma center within one posttraumatic hour, were enrolled in our prospective study. sNGAL-levels were assessed at admission (initial) and on day 2 after trauma. AKI was diagnosed by an increase of serum creatinine (sCr) level of at least 0.3 mg/dl within 48 hours.

**Results:**

Out of 30 men and 10 women (mean age, 43 years; mean ISS, 29), seven patients developed AKI, four of them needing RRT. AKI was diagnosed in 86% of the affected individuals until day 2. Day2-sNGAL-levels were higher in the AKI-group, compared to the no-AKI-group (p=0.049), and in patients treated with RRT than in individuals not needing RRT (p=0.037). Noteworthy, in patients not needing RRT sNGAL-levels significantly decreased from initial to day2-measurement (p=0.040). Furthermore, at any time point during our observation period polytraumatized patients with AKI and day2-sNGAL-levels of at least 181.0 ng/mL presented with higher sCr-levels compared to polytraumatized patients without AKI and day2-sNGAL-levels lower than 181.0 ng/mL (p≤0.029).

**Conclusion:**

In polytrauma victims suffering AKI an increase in sNGAL-level from initial to day2-assessment may signalize deterioration in kidney function and thus indicate AKI progression. Unlike initial sNGAL-levels day2-sNGAL-levels might be an appropriate tool to define AKI and to signify the need of RRT in polytraumatized patients.

## 1. Introduction

Acute kidney injury (AKI) is vaguely characterized by a sudden and rapid decline in kidney function resulting from renal cellular damage. Reduction in the glomerular filtration rate is the hallmark of AKI [1, 2]. It is characterized by an abrupt increase in nitrogen waste products, such as blood-urea nitrogen (BUN) and serum creatinine (sCr), and potentially by a reduced urine output [3]. In general, AKI is associated with an increase in morbidity and mortality, length of stay (LOS) at the hospital and at the intensive care unit (ICU), and in hospital costs [4]. It has been reported in 23.8% of critically ill adult trauma patients (mean Injury Severity Score (ISS), 19); their mortality rate was significantly higher compared to the no-AKI-group (24.4% versus 2.3%; p<0.0001) [5]. If AKI is not diagnosed and managed in a timely fashion, it may result in irreversible damage and furthermore in poor outcome [6]. Unfortunately, at present no effective curative therapy is available for AKI patients; the current treatment options are mainly supportive [7]. Although renal replacement therapy (RRT) represents a cornerstone in the management of severe AKI, several aspects of RRT including the appropriate timing of its initiation and completion remain still controversial [7–9].

Current definitions of AKI focus on impairment (loss of function) disregarding structural damage. However, concerns exist about using sCr as the standard parameter, as it is known to be insensitive to acute changes. Furthermore, sCr-levels can vary widely depending on age, gender, muscle mass, diet, medications, and hydration status [10].

NGAL is a small glycoprotein, which belongs to the lipocalin family. It has the ability to bind small lipophilic substances such as bacterial derived formyl peptides and lipopolysaccharides [11]. Human NGAL was originally identified as a novel protein isolated from secondary granules of neutrophils [12, 13]. It is expressed at low concentrations in healthy human tissues such as kidney, trachea, lung, stomach, small intestine, and colon [14], but its synthesis may be markedly upregulated in case of epithelial cell injury in the colon, liver, and lung, and especially in the kidney [15–19], as well as in malignancies of the breast, lung, colon, and pancreas [20–22]. NGAL exists in three different molecular forms (as a 25-kDa monomer, 45-kDa homodimer, or 135-kDa heterodimer covalently conjugated with gelatinase) in blood and urine [23]. Injured kidney epithelial cells predominantly secrete monomeric and, to some extent, heterodimeric NGAL, whereas neutrophils mainly release the homodimeric and, to some extent, the monomeric form [24, 25]. NGAL-levels assessed in the plasma of healthy adults range from 28.7 ng/mL (95% confidence interval, 26.4-33.2 ng/mL) to 167.0 ng/mL (95% confidence interval, 154.5-181.0 ng/mL) [26]. Itenov et al. have reported that NGAL-levels measured in serum and plasma cannot be directly compared as plasma levels were lower than serum levels [27].

Haase-Fielitz et al. [28] identified several studies strongly supporting the use of serum NGAL (sNGAL) and urinary NGAL (uNGAL) as a biomarker for the prediction of AKI in the setting of cardiac surgery, critical illness, and kidney transplantation. To our knowledge only one study focused on NGAL as a predictor of AKI in multiple injured patients. Makris et al. [29] identified uNGAL, assessed within 24 hours after injury, as a highly sensitive (91%) and specific (96%) predictor of early AKI (diagnosed within the first five posttraumatic days) in adult polytrauma victims.

Unfortunately, the likelihood of recovery from evolving AKI is difficult to estimate and this complicates the decision to initiate RRT as well as when to stop it [7]. Due to the fact that changes in the concentration of uNGAL might be induced by fluid resuscitation and diuretic therapy [30], we focused on sNGAL. We hypothesized that sNGAL may act as a diagnostic biomarker of early AKI in polytrauma victims, especially considering patients needing RRT.

## 2. Material and Methods

For this prospective observational study, which was approved by the local ethics committee, we enrolled 40 consecutive polytraumatized patients (ISS ≥ 16; age ≥ 18 years) with concomitant thoracic trauma (Abbreviated Injury Scale (AIS) Thorax ≥ 1), (1) who were directly admitted to our level I trauma center within one hour after the trauma occurred; (2) who were transferred to the intensive care unit (ICU) after initial treatment; (3) who survived for at least 48 hours; and (4) who gave their written consent. Following Makris et al. [29] individuals presenting with initial sCr-levels > 1.5 mg/dl were excluded. This cut-off value has been used to define renal impairment since the 1990s [5, 31–33].

Blood samples were collected upon admission and on day 2 from all participants, in serum separator tubes containing gel (Vacuette® 4 ml; Greiner Bio-One International). Subsequently samples were centrifuged at 3000 g for 15 minutes at room temperature and serum was aliquoted in cryovials and stored at −80°C until tested. Early AKI was defined by means of sCr-levels, which were routinely assessed daily within the first five posttraumatic days. Because baseline sCr-levels (prior to the trauma) were not available in our patient population and the urinary output criterion has proven to be too permissive [34], AKI was solely diagnosed by an increase of sCr-level higher than or equal to 0.3 mg/dl within 48 hours, (abbreviated as sCr-criterion in the following text) according to the “Kidney Disease Improving Global Outcomes” (KDIGO) Definition [35]. Of the patients suffering AKI, RRT (hemofiltration using citrate as anticoagulant) was indicated for those individuals, who presented with extensive muscle damage, indicated by significantly increased levels of creatine kinase and of myoglobin [36, 37], and who were additionally diagnosed with anuria and metabolic acidosis. A Human Lipocalin-2/NGAL Immunoassay Quantikine® ELISA Kit RD-DLCN20, Quantikine R&D-Systems, was used for NGAL-level measurement. All samples were analyzed in triplets and the mean values were calculated. According to the results presented in the literature [26, 27] the upper limit of the reference range of sNGAL was set at 181.0 ng/mL (sNGAL-criterion). Polytraumatized patients with sNGAL-levels lower than 181.0 mg/mL were denoted sNGAL-negative, whereas polytraumatized patients with sNGAL-levels of at least 181.0 mg/mL were termed sNGAL-positive.

Initially, an a priori power analysis by means of GPower 3.1.17 [38] estimated a patient number of 40 as the minimum sample size for detecting a large effect (effect size, 0.8), assuming a probability of 0.05 for *α* error and a probability of 0.25 for *β* error. The allocation ratio was set to 2 in accordance with the percentage of trauma victims suffering AKI as revealed by Brandt et al. [5] and Makris et al. [29]. Statistical analysis was performed using IBM SPSS Statistics Version 23. Parameters are presented as median and interquartile range in round brackets. Two continuous parameters were compared by means of the Mann-Whitney-Wilcoxon rank-sum test for unrelated samples. To compare four groups we performed Kruskal–Wallis tests. In case of significant differences Dunn tests were used as post hoc tests for pairwise comparison; p-values were adjusted by the Bonferroni correction for multiple testing. The Spearman rank coefficient r was calculated to indicate correlations. In general, significance was assumed at a p < 0.05. Statistical power 1-*β* was calculated in case of significant differences in sNGAL-levels.

## 3. Results

**Figure 1:**
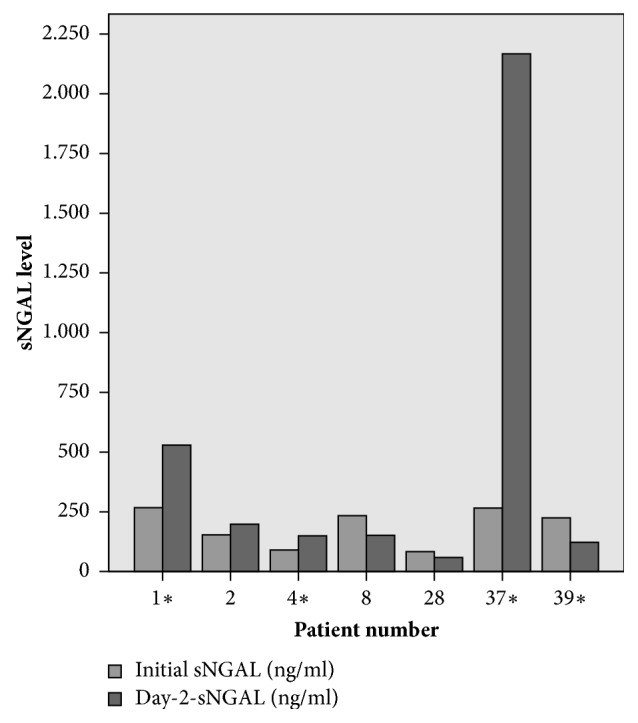
Initial and day2-sNGAL-levels in polytraumatized patients suffering AKI. Individuals needing RRT are marked by asterisks.

Thirty men and 10 women with a median age of 43 years [range, 18-77 years], a median ISS of 29 [range, 18-59], and a median AIS_Thorax_ of 4 [range, 1-5] met our inclusion criteria. AKI developed in seven patients (17.5%), six men and one woman, four of them requiring RRT. Initial and day2-sNGAL-levels are graphically displayed in Figure 1.

Of the individuals suffering AKI and not needing RRT, one was diagnosed with AKI on day 1 and two on day 2. Of the individuals undergoing RRT, three were diagnosed with AKI on day 1 and one on day 3. RRT was applied for 5.5 (2-14) days. One polytrauma victim (needing RRT) died after a stay of 24 days at the ICU due to multiple organ failure. Both age and ISS value were comparable in the AKI- and no-AKI-group (Table 1).

Whereas sCr- and BUN-levels significantly differed at admission in both groups, a barely significant difference in sNGAL-levels was detected only on day 2 (1-*β*=0.798).  Table 2 displays significant differences in ISS values and day2-sNGAL-levels (1-*β*=0.986) as well as in initial sCr- and initial BUN-levels between polytrauma victims needing and not needing RRT. Noteworthy, in patients not needing RRT sNGAL-levels significantly decreased from initial to day2-measurement (p=0.040). The increase in NGAL-levels in the RRT-group, however, could not reach statistical significance (p=0.273).

Significant weak and moderate correlations according to Spearman were calculated between the two sNGAL-levels and between the sNGAL-level assessed on day 2 and all sCr-levels except day 5 (Table 3).

Of interest, LOS [39 (21-63) days versus 26 (15.3-46) days] and ICU LOS [18 (3-24) days versus 6 (3.5-16) days] did not significantly differ in patients with/without AKI (p≥0.277). However, in individuals needing RRT, ICU LOS was significantly longer than in individuals not needing RRT [ICU LOS, 24 (19.5-42.8) days versus 6 (3.5-15.8) days, p=0.002; LOS, 45 (27.8-68) days versus 25 (15-47) days, p=0.195].

Although sCr-changes are widely accepted as a key component in all AKI consensus definitions, our data raise doubts about the unrestricted suitability of the sCr-criterion. A definition based on sNGAL-levels with a cut-off value of 181.0 ng/mL might be a promising alternative to define AKI, covering both impairment and structural damage. As a first step we subdivided our patients into group G1 (sNGAL-positive) combining individuals presenting with day2-sNGAL-levels of at least 181.0 ng/mL and into group G0 (sNGAL-negative) including all individuals with day2-sNGAL-levels lower than 181.0 ng/mL.  Table 4 reveals significant differences in sCr-levels between G0 and G1, starting at day 1 of our observation period.

Taking the sCr-criterion into account (sCr-positive denotes meeting the criterion), we formed four subgroups (G11, sNGAL-positive and sCr-positive; G10, sNGAL-positive and sCr-negative; G01, sNGAL-negative and sCr-positive; G00, sNGAL-negative and sCr-negative). Of interest, neither age nor ISS value was significantly different between any two of the four subgroups. The group-dependent sCr-levels and significant differences revealed by group comparisons are presented in Table 5.

Remarkably, significant differences in sCr-levels were revealed between G11 and G00 at any time point during our observation period. Finally, Table 6 is presented because it contains patient-specific information that is indispensable for comparative assessment of the sCr- and the sNGAL-criterion with regard to their capacity to identify those polytraumatized patients, who had sustained substantial renal cellular damage and/or a considerable decline in kidney function.

## 4. Discussion

Our prospective observational study revealed significant differences solely for day2-sNGAL-levels. They were higher in polytraumatized patients suffering AKI compared to the no-AKI-group and in polytraumatized patients treated with RRT than in individuals not needing RRT.

Furthermore, sNGAL-levels significantly decreased from initial to day2-assessment in polytraumatized patients not needing RRT. Finally, at any time point during our observation period sCr-levels were significantly higher in polytraumatized patients meeting both the sCr-criterion and the sNGAL-criterion than in polytraumatized patients not meeting both criteria.

Studies in humans and animal models have revealed a significant causal effect of AKI on extrarenal organ dysfunction [39–42]. In a murine model Grigoryev et al. [43] provided evidence that AKI leads to intrarenal inflammation that promotes organ inflammation in the lung. Global gene expression profiling revealed inflammation-associated transcriptional changes in both kidney and lung tissues with marked similarity. Thirty-one highly AKI-upregulated proinflammatory genes, including the Lcn2 gene, which codes for NGAL, were identified. These genes are supposed to directly leak from injured kidney tissue into the circulation, thus triggering and sustaining systemic inflammation.

Experimental and clinical evidence indicates that NGAL accumulates within two distinct pools, namely, a renal and a systemic pool [44, 45]. In patients suffering AKI NGAL is abundantly synthesized in the distal nephron, which comprises the renal pool, and rapidly secreted into the urine, accounting for the major fraction of uNGAL [16, 30, 46], whereas it is not efficiently introduced into the circulation [35]. Furthermore, AKI causes dramatically increased NGAL synthesis in distant organs, particularly in the liver and the lung [47], followed by its release into the circulation that constitutes the systemic pool [43]. Additional contributions to the systemic pool may derive from the fact that NGAL is an acute-phase reactant and may be released from neutrophils [30, 45] and macrophages [48]. sNGAL originates predominately from the systemic pool. Only a small amount is derived from the renal pool via backleak of glomerular filtrate across damaged tubular epithelium [49]. Furthermore, any decrease in glomerular filtration rate leads to a decrease in renal clearance of sNGAL, resulting in its further accumulation in the systemic pool [30, 50]. In normal conditions, sNGAL is freely filtered through the glomerular membrane and almost completely reabsorbed by endocytosis in the proximal tubule [45]. In AKI, however, a fraction of sNGAL may escape reabsorption due to proximal tubular injury, resulting in minimal uNGAL-levels [51].

Comorbidities and patient-specific parameters may constitute important confounders, when sNGAL-levels are interpreted in individuals suffering AKI [23]. Hence, children are the most suitable patients for investigating the course of sNGAL-levels referring to AKI. Mishra et al. [52] studied a homogenous population of 71 children with no comorbidities, undergoing cardiopulmonary bypass. In 20 children, who developed AKI, sNGAL-levels substantially rose after surgery, reaching the peak approximately two hours after AKI occurred, followed by a lesser but sustained significant increase versus baseline over the entire study period (5 days).

In polytrauma victims, tissue damage caused by the initial traumatic impact leads to a cascade of inflammatory immune responses, including neutrophils that form the most abundant cellular component of the host immune system [6]. Depending on injury pattern and severity, these neutrophils may substantially contribute to the systemic pool of NGAL, thus overlapping the increase in sNGAL-level provoked by AKI. NGAL is synthesized during maturation of granulocyte precursors in the bone marrow and stored in specific granules of mature neutrophils [45]. In the blood of healthy adults neutrophils are in a resting state [53], which ensures that their toxic intracellular contents are not accidentally released to damage host tissue [54]. Throughout the body neutrophils are distributed about equally between two pools, the circulating pool and the marginating pool [55], which is located predominantly within the liver, spleen, and bone marrow [56]. Marginated neutrophils may be rapidly mobilized during an inflammatory episode or in response to infection, resulting in a dramatic rise in the number of circulating neutrophils [57]. They become primed by agents such as TNF-*α*, IL-8, and IFN-*γ* and are then recruited into the site of injury within 10 minutes after the trauma occurred [58], where they encounter activating signals [54]. Thereafter, they inter alia release their granular contents (including NGAL) into the circulation [13, 59], thus forming the first line of cellular defense against infections caused by bacterial and fungal pathogens [60, 61]. Neutrophil infiltration has been reported to peak at 24 hours after the triggering event [62, 63]. Provided that the inflammatory causes are largely cleared, it is then promptly ceased in order to confine tissue damage [64].

As NGAL-synthesis triggered by AKI and NGAL-release after neutrophil activation starts and peaks at different times according to the results presented in the literature, sNGAL-levels have to be interpreted time dependently. At initial assessment, taken not later than about one hour after injury, the release of sNGAL by activated neutrophils has already started, whereas sNGAL-levels originating from AKI are far from reaching their peak. In view of the facts we hypothesize that the initial sNGAL-levels mainly reflect neutrophilic inflammation and overall injury severity. Thus, initial sNGAL-levels, assessed immediately after hospital admission, are not suitable for early AKI diagnosis in polytraumatized patients. AKI, however, has to be considered as the primary source of day2-sNGAL-levels. While sNGAL-levels originating from AKI still significantly increased versus baseline at that time, sNGAL-release by activated neutrophils already decreased. This presumption is confirmed by the fact that higher sNGAL-levels could be observed in the AKI-group on day 2 and that a weak or moderate correlation could be calculated only between the day2-sNGAL-level and the sCr-levels assessed on day 0 to day 4 after the trauma occurred. Due to the fact that day2-sNGAL-levels were markedly increased in individuals needing RRT compared to patients who did not receive RRT, day2-sNGAL-levels might indicate its need in polytraumatized patients. Moreover, sNGAL may serve as a progression marker as suggested by the different course of sNGAL-levels in the RRT- and no-RRT-group (increasing vs. decreasing values). Due to the fact that RRT does not substantially influence sNGAL concentration [65], a further rise of sNGAL-level may signalize deterioration in kidney function and predict AKI progression.

Although sCr-level increase is widely accepted as a reliable tool for AKI definition, the question arises if day2-sNGAL-levels might be better suited to fulfill this task as they reflect the injury leading to renal impairment. A subdivision of our polytraumatized patients according to our sNGAL-criterion resulted in higher sCr-levels in G1, the day2-sNGAL-positive-group, starting on day 1 (Table 4). This finding indicates a rapid decline in kidney function provoked by renal cellular damage in G1. G0 and G1 were further subdivided according to the sCr-criterion. As Table 5 reveals, 31 patients were classified identically by the sCr- and the sNGAL-criterion, namely, positive in three individuals (G11) and negative in 28 individuals (G00). However, G01 and G10 formed a borderline group, combining nine polytraumatized patients (eight males and one female), who have to be separately analyzed as the severity of renal structural damage does not coincide with the degree of renal functionality. Patients 4, 8, 28, and 39 of the borderline group presented with day2-sNGAL-levels lower than 181.0 ng/mL. Female patient 28 is characterized by an initial sNGAL-level of 83.9 ng/mL, which was considerably lower than the upper limit of the reference range [26] and declined to 58.9 ng/mL on day 2. As all but one sCr-level were within the reference range (0.48-0.93 mg/dL) for women [65], she must not be diagnosed with AKI in our opinion, even though there was an sCr-increase of 0.3 ng/mL. As RRT was needed in patients 4 and 39, it is evident that both suffered from AKI. Finally, in patient 8 an increase in sCr-level of 40.5% from day 0 to day 1 was observed, which was almost three times higher than the Reference Change Value of 15.03% for sCr [66], thus mirroring a pathological process and the presence of AKI. Patients 16, 20, 26, 30, and 34 of the borderline group were classified day2-sNGAL-positive and sCr-negative. Due to the fact that an increase in sCr-level of 24.4% from day 0 to day 1 was detected in patient 16 and that the sCr-levels of patient 34 exceed the reference range for men (0.63-1.16 mg/dL) [65] on three days, both could have suffered AKI. The high day2-sNGAL-levels that patients 20, 26, and 30 have in common indicate substantial renal cellular damage, which surprisingly did not impact sCr-levels and thus kidney function within the first five posttraumatic days. Since nowadays AKI is the descriptive term for the clinical condition that occurs, when the renal excretory function is critically and acutely decreased [67], those patients did not suffer early AKI given the current definition. Nevertheless, any negative impact on cellular level cannot be ruled out in their cases.

In summary, three patients (4, 8, 39) with minor cellular renal damage and impaired kidney function were detected, whereas three patients (20, 26, 30) presented with substantial renal cellular damage, which did not manifest in functional deterioration at least within the first five posttraumatic days. Thus, a definition of acute kidney injury by means of the day2-sNGAL-criterion (based on an empirically determined cut-off value) in polytraumatized patients would primarily identify individuals sustaining renal cellular damage, which provokes impaired renal function in most cases. Applying the sCr-criterion to day2-sNGAL-negative patients would minimize the number of false-negative cases.

Limitations of our study include the fact that the number of enrolled patients only met minimum requirement and in consequence was too small to perform meaningful subgroup analysis focusing on injury pattern and severity. However, by restricting our study population to polytrauma victims suffering thoracic injury we have accounted for the particular role of the lung, which has to be considered as a potential major source of NGAL provoked by both AKI and traumatic alveolar epithelial injury. Finally, with regard to day2-sNGAL-levels, we referred to a reference range measured in plasma, although plasma levels are supposed to be lower than serum levels [27]. In order to compensate this difference we chose 181.0 ng/mL (upper limit of the reference range, 167.0 ng/mL; 95% confidence interval, 154.5-181.0) as our cut-off value.

## 5. Conclusions

Our findings suggest that sNGAL-levels, assessed not until day 2 after the trauma occurred, may serve as a reliable marker of structural kidney injury and AKI progression in polytrauma victims, thus indicating the need and duration of RRT. Furthermore, our data point out that day2-sNGAL-levels might provide a sound basis for a novel definition of AKI, covering both renal structural damage and impairment, if reference ranges are empirically determined in major studies. This may then contribute further to determine the optimal individual treatment regimen in a timely manner.

## Figures and Tables

**Table 1:** Differences according to the presence of AKI.

	AKI	No-AKI	p-value
Age (years)	**56.0 (53.0-70.0)**	**37.0 (26.0-54.5)**	**0.008**
ISS	34 (19-41)	29 (23-36)	0.927
sNGAL initial (ng/mL)	224.4 (90.5-266.1)	142.7 (71.1-203.0)	0.192
sNGAL Day 2 (ng/mL)	**151.9 (122.2-529.3)**	**104.3 (72.7-152.9)**	**0.049**
sCr initial (mg/dL)	**1.37 (0.94-1.48)**	**1.00 (0.83-1.17)**	**0.037**
BUN initial (mg/dL)	**22.50 (13.20-24.50)**	**14.60 (11.30-17.70)**	**0.044**

Significant differences are highlighted in bold letters.

**Table 2:** Differences according to the need of RRT.

	RRT	No-RRT	p-value
Age (years)	55.5 (42.3-59.0)	41.0 (28.5-55.0)	0.207
ISS	**38 (34-43)**	**27 (21-34)**	**0.042**
sNGAL initial (ng/mL)	245.3 (124.0-266.9)	143.5 (77.5-203.8)	0.148
sNGAL Day 2 (ng/mL)	**339.6 (129.1-1757.6)**	**106.3 (69.3-155.4)**	**0.037**
sCr initial (mg/dL)	**1.48 (1.07-1.49)**	**1.01 (0.83-1.17)**	**0.021**
BUN initial (mg/dL)	**22.90 (18.45-24.20)**	**14.50 (11.43-17.70)**	**0.009**

Significant differences are highlighted in bold letters.

**Table 3:** Correlations between biomarker levels, age, and ISS.

	sNGAL initial	sNGAL Day 2	BUN initial	sCr initial	sCr Day 1	sCr Day 2	sCr Day 3	sCr Day 4	sCr Day 5	Age	ISS
sNGAL initial											
Spearman *ρ*		**0.335**	0.063	0.249	0.262	0.096	0.092	0.201	0.226	0.101	-0.043
p-value		**0.035**	0.698	0.122	0.102	0.555	0.574	0.214	0.160	0.543	0.791
sNGAL Day 2											
Spearman *ρ*	**0.335**		0.124	**0.382**	**0.458**	**0.414**	**0.352**	**0.332**	0.300	0.240	0.078
p-value	**0.035**		0.447	**0.015**	**0.003**	**0.008**	**0.026**	**0.036**	0.060	0.136	0.633

Significant correlations are highlighted in bold letters.

**Table 4:** Differences in sCr-levels according to group assignment.

	sCr	sCr	sCr	sCr	sCr	sCr
	initial	Day 1	Day 2	Day 3	Day 4	Day 5
	(mg/dL)	(mg/dL)	(mg/dL)	(mg/dL)	(mg/dL)	(mg/dL)
G1 n=8	1.19	1.17	1.12	1.10	1.09	1.06
	(0.97-1.45)	(0.97-1.68)	(0.98-1.66)	(0.88-1.54	(0.78-1.47)	(0.85-1.44)
G0 n=32	1.00	0.97	0.91	0.83	0.77	0.80
	(0.82-1.17)	(0.78-1.10)	(0.79-1.00)	(0.70-0.92)	(0.68-0.87)	(0.65-0.90)
	*p=0.055*	*p=0.027*	*p=0.007*	*p=0.004*	*p=0.011*	*p=0.009*

**Table 5:** Differences in sCr- levels according to group assignment.

	sCr initial (mg/dL)	p-value	sCr Day 1 (mg/dL)	p-value	sCr Day 2 (mg/dL)	p-value	sCr Day 3 (mg/dL)	p-value	sCr Day 4 (mg/dL)	p-value	sCr Day 5 (mg/dL)	p-value
G11 n=3	1.48 (1.37-1.48)	G11-G00 *0.029*	1.75 (1.45-1.75)	G11-G00 *0.021*	1.82 (1.18-1.82)	G11-G00 *0.012* G01-G00 *0.021*	1.57 (1.45-1.57)	G11-G00 *0.008*	1.53 (1.28-1.53)	G11-G00 *0.011*	1.48 (1.17-1.48)	G11-G00 *0.015*
G10 n=5	1.06 (0.90-1.19)		1.07 (0.93-1.17)		1.02 (0.91-1.12)		0.95 (0.82-1.10)		0.84 (0.74-1.09)		0.93 (0.78-1.13)	
G01 n=4	1.03 (0.86-1. 38)		1.41 (0.87-2.20)		1.18 (1.07-1.71)		0.93 (0.91-1.31)		0.90 (0.84-1.29)		0.93 (0.88-1.32)	
G00 n=28	1.00 (0.82-1.17)		0.96 (0.78-1.06)		0.89 (0.78-0.94)		0.80 (0.69-0.91)		0.74 (0.68-0.85)		0.78 (0.64-0.89)	
	*p=0.046*		*p=0.010*		*p=0.001*		*p=0.002*		*p=0.004*		*p=0.005*	

**Table 6:** Patient-specific demographic data and biomarker levels.

**Nr.**	**Gender**	**Age**	**ISS**	**AIS** _**Thorax**_	**AIS** _**Abdo-men**_	**AIS** _**Head**_	**NGAL** **Initial** ** (ng/mL)**	**NGAL** **Day 2 (ng/mL)**	**Acute surgery**	**BUN** **initial (mg/dL)**	**Scr** **initial (mg/dL)**	**Scr** **Day 1 (mg/dL)**	**Scr** **Day 2 (mg/dL)**	**Scr** **Day 3 (mg/dL)**	**Scr** **Day 4 (mg/dL)**	**Scr** **Day 5 (mg/dL)**	**AKI**	**RRT**	**Sub-group**	**Fata-lity**
1	m	56	43	5	0	0	267.1	529.3	yes	22.50	1.49	1.75	1.18	1.57	1.53	1.48	yes	yes	G11	no
2	m	70	19	3	0	0	153.6	198.3	yes	28.00	1.37	1.45	1.82	1.45	1.28	1.17	yes	no	G11	no
3	m	51	29	2	0	0	66.7	65.9	no	11.30	0.72	0.71	0.65	0.70	0.70	0.65	no	no	G00	no
4	m	60	34	4	2	2	90.5	149.8	yes	17.10	0.94	1.25	1.09	0.92	0.93	0.95	yes	yes	G01	no
5	m	34	29	4	2	2	240.5	79.5	yes	20.20	1.17	0.98	0.94	1.19	1.21	1.01	no	no	G00	no
6	f	60	18	3	0	0	229.1	156.6	yes	15.90	1.21	1.26	1.13	1.06	0.98	0.95	no	no	G00	no
7	m	63	17	4	0	0	64.9	63.2	no	22.20	1.26	1.20	1.22	1.20	1.21	1.20	no	no	G00	no
8	m	53	20	4	0	0	233.9	151.9	yes	13.20	1.11	1.56	1.27	0.94	0.87	0.87	yes	no	G01	no
9	m	34	24	4	0	0	194.4	135.6	no	14.60	0.98	0.96	0.89	0.82	0.86	0.85	no	no	G00	no
10	m	33	38	2	5	5	144.3	104.3	no	15.00	1.14	1.04	0.89	0.73	0.75	0.64	no	no	G00	no
11	f	19	24	3	0	0	96.6	169.8	yes	13.70	0.83	0.64	0.86	0.73	0.68	0.62	no	no	G00	no
12	f	36	22	3	0	0	105.0	79.6	yes	7.80	0.69	0.63	0.62	0.56	0.63	0.61	no	no	G00	no
13	f	35	50	5	2	2	128.5	102.2	no	10.90	1.30	0.94	0.78	0.64	0.62	0.72	no	no	G00	no
14	m	24	43	3	2	2	142.7	92.9	yes	12.20	1.16	1.08	0.83	0.84	0.79	0.90	no	no	G00	no
15	m	43	29	4	3	3	201.4	100.4	no	13.60	0.95	0.89	0.92	0.91	0.80	1.02	no	no	G00	no
16	m	43	22	1	0	0	45.3	238.7	yes	9.40	0.86	1.07	0.96	0.85	0.71	0.73	no	no	G10	no
17	m	24	24	2	0	0	152.1	108.2	no	13.40	1.00	0.90	0.89	0.81	0.85	0.89	no	no	G00	no
18	m	52	43	5	0	0	263.3	149.1	yes	5.80	0.82	1.02	0.82	0.64	0.68	0.65	no	no	G00	no
19	f	18	38	5	3	3	155.0	35.2	yes	9.90	0.64	0.84	0.77	0.68	0.64	0.57	no	no	G00	no
20	m	52	26	4	0	0	596.1	193.7	yes	11.30	1.06	0.93	0.85	0.79	0.84	0.82	no	no	G10	no
21	f	39	27	3	0	0	204.6	123.4	yes	17.70	0.83	0.65	0.51	0.53	0.49	0.49	no	no	G00	no
22	m	22	34	3	4	4	51.9	56.3	yes	18.50	0.69	0.88	0.86	0.74	0.70	0.64	no	no	G00	no
23	m	22	27	3	0	0	151.8	93.6	yes	21.50	1.04	0.99	0.91	0.85	0.85	0.83	no	no	G00	no
24	f	31	17	3	0	0	159.8	42.3	no	15.50	0.79	0.75	0.72	0.69	0.69	0.67	no	no	G00	no
25	m	22	29	3	1	1	75.4	139.0	yes	18.10	1.46	1.00	0.94	1.07	0.88	0.89	no	no	G00	no
26	m	30	59	5	5	5	121.6	250.3	yes	9.30	1.25	1.13	1.15	0.99	1.03	0.94	no	no	G10	no
27	m	60	45	5	3	3	115.5	139.3	yes	25.10	1.28	1.35	1.10	0.89	0.85	0.79	no	no	G00	no
28	f	77	17	1	2	2	83.9	58.9	yes	11.80	0.83	0.74	1.06	0.91	0.83	0.90	yes	no	G01	no
29	m	28	21	4	0	0	269.3	123.2	no	15.20	0.91	0.74	0.67	0.71	0.67	0.81	no	no	G00	no
30	m	61	29	3	0	0	174.5	224.8	no	13.10	0.94	0.92	1.02	0.95	0.76	0.93	no	no	G10	no
31	m	67	24	2	0	0	47.9	120.3	yes	17.50	0.73	0.82	0.78	0.65	0.58	0.59	no	no	G00	no
32	m	54	25	3	0	0	40.2	53.8	yes	15.60	1.13	0.95	0.90	0.90	0.94	0.89	no	no	G00	no
33	f	70	17	2	2	2	320.3	91.1	no	14.40	1.02	1.10	0.94	0.76	0.70	0.65	no	no	G00	no
34	m	55	29	4	0	0	207.9	186.0	yes	13.50	1.12	1.20	1.08	1.20	1.14	1.32	no	no	G10	no
35	m	51	21	4	0	0	131.1	168.6	no	18.80	1.08	1.06	0.91	0.79	0.70	0.76	no	no	G00	no
36	f	37	29	5	0	0	133.0	42.1	yes	15.00	1.17	1.13	0.94	0.85	0.83	0.78	no	no	G00	no
37	m	55	34	4	3	3	266.1	2167.0	yes	24.50	1.48	2.09	2.06	2.11	2.52	2.28	yes	yes	G11	yes
38	m	22	34	4	0	0	43.9	101.7	yes	17.70	1.00	0.99	0.92	1.06	0.74	0.78	no	no	G00	no
39	m	38	41	4	2	2	224.4	122.2	yes	23.30	1.47	2.41	1.86	1.43	1.41	1.44	yes	yes	G01	no
40	m	55	41	4	2	2	57.1	45.1	yes	8.50	0.82	0.76	1.02	0.91	0.74	1.02	no	no	G00	no

## Data Availability

The data used to support the findings of this study are included within the article.

## Abbreviations

AIS: Abbreviated Injury Scale
AKI: Acute kidney injury
BUN: Blood-urea nitrogen
ISS: Injury Severity Score
ICU: Intensive care unit
LOS: Length of stay
RRT: Renal replacement therapy
sCr: Serum creatinine
sNGAL: Serum neutrophil gelatinase-associated lipocalin
uNGAL: Urinary NGAL.

## References

[1] Molitoris B. A. (2012). Measuring glomerular filtration rate in acute kidney injury: Yes, but not yet. *Critical Care*.

[2] Bellomo R., Ronco C., Kellum J. A., Mehta R. L., Palevsky P. (2004). Acute renal failure—definition, outcome measures, animal models, fluid therapy and information technology needs: the Second International Consensus Conference of the Acute Dialysis Quality Initiative (ADQI) Group. *Critical Care*.

[3] Rahman M., Shad F., Smith MC. (2012). Acute kidney injury: a guide to diagnosis and management. *American Family Physician*.

[4] Hobson C., Ozrazgat-Baslanti T., Kuxhausen A. (2015). Cost and mortality associated with postoperative acute kidney injury. *Annals of Surgery*.

[5] Brandt M.-M., Falvo A. J., Rubinfeld I. S., Blyden D., Durrani N. K., Horst H. M. (2007). Renal dysfunction in trauma: Even a little costs a lot. *Journal of Trauma - Injury Infection and Critical Care*.

[6] Mehta R. L., Cerdá J., Burdmann E. A. (2015). International society of nephrology's 0 by 25 initiative for acute kidney injury (zero preventable deaths by 2025): a human rights case for nephrology. *The Lancet*.

[7] Ronco C., Ricci Z., De Backer D. (2015). Renal replacement therapy in acute kidney injury: controversy and consensus. *Critical Care*.

[8] Negi S., Koreeda D., Kobayashi S., Iwashita Y., Shigematu T. (2016). Renal replacement therapy for acute kidney injury. *Renal Replacement Therapy*.

[9] Bhatt GC., Das RR. (2017). Early versus late initiation of renal replacement therapy in patients with acute kidney injury-a systematic review meta-analysis of randomized controlled trials. *BMC Nephrol*.

[10] Kanagasundaram N. S. (2015). Pathophysiology of ischaemic acute kidney injury. *Annals of Clinical Biochemistry*.

[11] Cowland J. B., Borregaard N. (1997). Molecular characterization and pattern of tissue expression of the gene for neutrophil gelatinase-associated lipocalin from humans. *Genomics*.

[12] Kjeldsen L., Bainton D. F., Sengeløv H., Borregaard N. (1994). Identification of neutrophil gelatinase-associated lipocalin as a novel matrix protein of specific granules in human neutrophils. *Blood*.

[13] Xu S. Y., Carlson M., Engstrom A., Garcia R., Peterson C. G. B., Venge P. (1994). Purification and characterization of a human neutrophil lipocalin (HNL) from the secondary granules of human neutrophils. *Scandinavian Journal of Clinical & Laboratory Investigation*.

[14] Xu S., Venge P. (2000). Lipocalins as biochemical markers of disease. *Biochimica et Biophysica Acta (BBA) - Protein Structure and Molecular Enzymology*.

[15] Supavekin S., Zhang W., Kucherlapati R., Kaskel F. J., Moore L. C., Devarajan P. (2003). Differential gene expression following early renal ischemia/reperfusion. *Kidney International*.

[16] Mishra J., Qing M. A., Prada A. (2003). Identification of neutrophil gelatinase-associated lipocalin as a novel early urinary biomarker for ischemic renal injury. *Journal of the American Society of Nephrology*.

[17] Devarajan P., Mishra J., Supavekin S., Patterson L. T., Potter S. S. (2003). Gene expression in early ischemic renal injury: Clues towards pathogenesis, biomarker discovery, and novel therapeutics. *Molecular Genetics and Metabolism*.

[18] Mishra J., Mori K., Ma Q., Kelly C., Barasch J., Devarajan P. (2004). Neutrophil gelatinase-associated lipocalin: a novel early urinary biomarker for cisplatin nephrotoxicity. *American Journal of Nephrology*.

[19] Mishra J., Mori K., Ma Q. (2004). Amelioration of ischemic acute renal injury by neutrophil gelatinase-associated lipocalin. *Journal of the American Society of Nephrology*.

[20] Nielsen B. S., Borregaard N., Bundgaard J. R., Timshel S., Sehested M., Kjeldsen L. (1996). Induction of NGAL synthesis in epithelial cells of human colorectal neoplasia and inflammatory bowel diseases. *Gut*.

[21] Stoesz S. P., Friedl A., Haag J. D., Lindstrom M. J., Clark G. M., Gould M. N. (1998). Heterogeneous expression of the lipocalin NGAL in primary breast cancers. *International Journal of Cancer*.

[22] Furutani M., Arii S., Mizumoto M., Kato M., Imamura M. (1998). Identification of a neutrophil gelatinase-associated lipocalin mRNA in human pancreatic cancers using a modified signal sequence trap method. *Cancer Letters*.

[23] Mårtensson J., Bellomo R. (2014). The rise and fall of NGAL in acute kidney injury. *Blood Purification*.

[24] Cai L., Rubin J., Han W., Venge P., Xu S. (2010). The origin of multiple molecular forms in urine of HNL/NGAL. *Clinical Journal of the American Society of Nephrology*.

[25] Mårtensson J., Xu S., Bell M., Martling C.-R., Venge P. (2012). Immunoassays distinguishing between HNL/NGAL released in urine from kidney epithelial cells and neutrophils. *Clinica Chimica Acta*.

[26] Makris K., Stefani D., Makri E. (2015). Evaluation of a particle enhanced turbidimetric assay for the measurement of neutrophil gelatinase-associated lipocalin in plasma and urine on Architect-8000: Analytical performance and establishment of reference values. *Clinical Biochemistry*.

[27] Itenov T. S., Bangert K., Christensen P. H., Jensen J., Bestle M. H. (2014). Serum and Plasma Neutrophil Gelatinase Associated Lipocalin (NGAL) Levels are Not Equivalent in Patients Admitted to Intensive Care. *Journal of Clinical Laboratory Analysis*.

[28] Haase-Fielitz A., Haase M., Devarajan P. (2014). Neutrophil gelatinase-associated lipocalin as a biomarker of acute kidney injury: a critical evaluation of current status. *Annals of Clinical Biochemistry*.

[29] Makris K., Markou N., Evodia E. (2009). Urinary neutrophil gelatinase-associated lipocalin (NGAL) as an early marker of acute kidney injury in critically ill multiple trauma patients. *Clinical Chemistry and Laboratory Medicine*.

[30] Devarajan P. (2010). Neutrophil gelatinase-associated lipocalin: a promising biomarker for human acute kidney injury. *Biomarkers in Medicine*.

[31] Taliercio C. P., Vlietstra R. E., Ilstrup D. M. (1991). A randomized comparison of the nephrotoxicity of iopamidol and diatrizoate in high risk patients undergoing cardiac angiography. *Journal of the American College of Cardiology*.

[32] Akhter M. W., Aronson D., Bitar F. (2004). Effect of elevated admission serum creatinine and its worsening on outcome in hospitalized patients with decompensated heart failure. *American Journal of Cardiology*.

[33] Chinen R., Câmara N. O. S., Nishida S. (2006). Determination of renal function in long-term heart transplant patients by measurement of urinary retinol-binding protein levels. *Brazilian Journal of Medical and Biological Research*.

[34] Md Ralib A., Pickering J. W., Shaw G. M., Endre Z. H. (2013). The urine output definition of acute kidney injury is too liberal. *Critical Care*.

[35] Pickering J. W., Endre Z. H. (2013). The definition and detection of acute kidney injury. *Journal of Renal Injury Prevention*.

[36] Strecker W., Gebhard F., Rager J., Brückner U. B., Steinbach G., Kinzl L. (1999). Early biochemical characterization of soft-tissue trauma and fracture trauma. *Journal of Trauma*.

[37] Hackl J. M., Neumann M., Weirather E., Stroschneider E. (1990). Myoglobin release and renal function in polytraumatized patients in intensive care. *Anaesthesist*.

[38] Faul F., Erdfelder E., Lang A., Buchner A. (2007). G^*^Power 3: a flexible statistical power analysis program for the social, behavioral, and biomedical sciences. *Behavior Research Methods*.

[39] Chen Y. C., Fang J. T., Tien Y. C., Chang M. Y., Huang C. C. (2000). Organ system failures predict prognosis in critically ill patients with acute renal failure requiring dialysis. *Changgeng yi xue za zhi / Changgeng ji nian yi yuan = Chang Gung medical journal / Chang Gung Memorial Hospital*.

[40] Hassoun H. T., Grigoryev D. N., Lie M. L. (2007). Ischemic acute kidney injury induces a distant organ functional and genomic response distinguishable from bilateral nephrectomy. *American Journal of Physiology-Renal Physiology*.

[41] Kelly K. J. (2003). Distant effects of experimental renal ischemia/reperfusion injury. *Journal of the American Society of Nephrology*.

[42] Shiao C., Wu P., Huang T. (2015). Long-term remote organ consequences following acute kidney injury. *Critical Care*.

[43] Grigoryev D. N., Liu M., Hassoun H. T., Cheadle C., Barnes K. C., Rabb H. (2008). The local and systemic inflammatory transcriptome after acute kidney injury. *Journal of the American Society of Nephrology*.

[44] Schmidt-Ott K. M., Mori K., Kalandadze A. (2006). Neutrophil gelatinase-associated lipocalin-mediated iron traffic in kidney epithelia. *Current Opinion in Nephrology and Hypertension*.

[45] Schmidt-Ott K. M., Mori K., Jau Y. L. (2007). Dual action of neutrophil gelatinase-associated lipocalin. *Journal of the American Society of Nephrology*.

[46] Helanova K., Spinar J., Parenica J. (2014). Diagnostic and prognostic utility of Neutrophil Gelatinase-Associated Lipocalin (NGAL) in patients with cardiovascular diseases - Review. *Kidney and Blood Pressure Research*.

[47] Shrestha K., Shao Z., Singh D., Dupont M., Tang W. H. W. (2012). Relation of systemic and urinary neutrophil gelatinase-associated lipocalin levels to different aspects of impaired renal function in patients with acute decompensated heart failure. *American Journal of Cardiology*.

[48] Jung M., Weigert A., Tausendschön M. (2012). Interleukin-10-induced neutrophil gelatinase-associated lipocalin production in macrophages with consequences for tumor growth. *Molecular and Cellular Biology*.

[49] Myers B. D., Chui F., Hilberman M., Michaels A. S. (1979). Transtubular leakage of glomerular filtrate in human acute renal failure.. *American Journal of Physiology-Endocrinology and Metabolism*.

[50] Schmidt-Ott K. M. (2011). Neutrophil gelatinase-associated lipocalin as a biomarker of acute kidney injury - Where do we stand today?. *Nephrology Dialysis Transplantation *.

[51] Mori K., Lee H. T., Rapoport D. (2005). Endocytic delivery of lipocalin-siderophore-iron complex rescues the kidney from ischemia-reperfusion injury. *The Journal of Clinical Investigation*.

[52] Mishra J., Dent C., Tarabishi R. (2005). Neutrophil gelatinase-associated lipocalin (NGAL) as a biomarker for acute renal injury after cardiac surgery. *The Lancet*.

[53] Miralda I., Uriarte S. M., McLeish K. R. (2017). Multiple phenotypic changes define neutrophil priming. *Frontiers in Cellular and Infection Microbiology*.

[54] Wright H. L., Moots R. J., Bucknall R. C., Edwards S. W. (2010). Neutrophil function in inflammation and inflammatory diseases. *Rheumatology*.

[55] Athens J. W., Haab O. P., Raab S. O. (1961). Leukokinetic studies. IV. The total blood, circulating and marginal granulocyte pools and the granulocyte turnover rate in normal subjects. *The Journal of Clinical Investigation*.

[56] Saverymuttu S. H., Peters A. M., Keshavarzian A., Reavy H. J., Lavender J. P. (1985). The kinetics of ^111^Indium distribution following injection of ^111^Indium labelled autologous granulocytes in man. *British Journal of Haematology*.

[57] Furze R. C., Rankin S. M. (2008). Neutrophil mobilization and clearance in the bone marrow. *The Journal of Immunology*.

[58] Hietbrink F., Koenderman L., Rijkers G. T., Leenen L. P. H. (2006). Trauma: the role of the innate immune system. *World Journal of Emergency Surgery*.

[59] Borregaard N., Cowland J. B. (1997). Granules of the human neutrophilic polymorphonuclear leukocyte. *Blood*.

[60] Sadik C. D., Kim N. D., Luster A. D. (2011). Neutrophils cascading their way to inflammation. *Trends in Immunology*.

[61] Neher M. D., Weckbach S., Flierl M. A., Huber-Lang M. S., Stahel P. F. (2011). Molecular mechanisms of inflammation and tissue injury after major trauma-is complement the "bad guy"?. *Journal of Biomedical Science*.

[62] Clausen F., Lorant T., Lewén A., Hillered L. (2007). T lymphocyte trafficking: A novel target for neuroprotection in traumatic brain injury. *Journal of Neurotrauma*.

[63] Cuello C., Wakefield D., Di Girolamo N. (2002). Neutrophil accumulation correlates with type IV collagenase/gelatinase activity in endotoxin induced uveitis. *British Journal of Ophthalmology*.

[64] Bian Z., Guo Y., Ha B., Zen K., Liu Y. (2012). Regulation of the inflammatory response: enhancing neutrophil infiltration under chronic inflammatory conditions. *The Journal of Immunology*.

[65] Pottel H., Vrydags N., Mahieu B., Vandewynckele E., Croes K., Martens F. (2008). Establishing age/sex related serum creatinine reference intervals from hospital laboratory data based on different statistical methods. *Clinica Chimica Acta*.

[66] Bugdayci G., Oguzman H., Arattan H. Y., Sasmaz G. (2015). The use of reference change values in clinical laboratories. *Clinical Laboratory*.

[67] Tögel F., Westenfelder C. (2014). Recent advances in the understanding of acute kidney injury. *F1000Prime Reports*.

